# Biodegradable Polymers

**DOI:** 10.3390/ma2020307

**Published:** 2009-04-01

**Authors:** Isabelle Vroman, Lan Tighzert

**Affiliations:** Groupe de Recherche En Sciences Pour l'Ingénieur (GRESPI), Laboratoire d'Etudes des Matériaux Polymères d'Emballage (LEMPE), Ecole Supérieure d'Ingénieurs en Emballage et Conditionnement (ESIEC), Esplanade Roland Garros - Pôle Henri Farman, BP 1029, 51686 Reims Cedex 2, France; E-Mail: lan.tighzert@univ-reims.fr

**Keywords:** Biodegradable polymers, polyesters, polyamides, polyurethanes, biopolymers, biodegradable polymer blends

## Abstract

Biodegradable materials are used in packaging, agriculture, medicine and other areas. In recent years there has been an increase in interest in biodegradable polymers. Two classes of biodegradable polymers can be distinguished: synthetic or natural polymers. There are polymers produced from feedstocks derived either from petroleum resources (non renewable resources) or from biological resources (renewable resources). In general natural polymers offer fewer advantages than synthetic polymers. The following review presents an overview of the different biodegradable polymers that are currently being used and their properties, as well as new developments in their synthesis and applications.

## 1. Introduction

The same durability properties which make plastics ideal for many applications such as in packaging, building materials and commodities, as well as in hygiene products, can lead to waste-disposal problems in the case of traditional petroleum-derived plastics, as these materials are not readily biodegradable and because of their resistance to microbial degradation, they accumulate in the environment. In addition in recent times oil prices have increased markedly. These facts have helped to stimulate interest in biodegradable polymers and in particular biodegradable biopolymers. Biodegradable plastics and polymers were first introduced in 1980s. There are many sources of biodegradable plastics, from synthetic to natural polymers. Natural polymers are available in large quantities from renewable sources, while synthetic polymers are produced from non renewable petroleum resources.

Biodegradation takes place through the action of enzymes and/or chemical deterioration associated with living organisms. This event occurs in two steps. The first one is the fragmentation of the polymers into lower molecular mass species by means of either abiotic reactions, i.e. oxidation, photodegradation or hydrolysis, or biotic reactions, i.e. degradations by microorganisms. This is followed by bioassimilation of the polymer fragments by microorganisms and their mineralisation. Biodegradability depends not only on the origin of the polymer but also on its chemical structure and the environmental degrading conditions. Mechanisms and estimation techniques of polymer biodegradation have been reviewed [1]. The mechanical behaviour of biodegradable materials depends on their chemical composition [2,3], the production, the storage and processing characteristics [4,5], the ageing and the application conditions [6].

## 2. Biodegradable Polymers Derived from Petroleum Resources

These are synthetic polymers with hydrolysable functions, such as ester, amide and urethane, or polymers with carbon backbones, in which additives like antioxidants are added. Recent developments in this area have been reported [7]. Synthesis, properties and biodegradability of the main classes and new families of synthetic polymers are discussed below.

### 2.1. Polymers with additives

Most conventional polymers derived from petroleum resources are resistant to degradation. To facilitate their biodegradation, additives are added. One method to degrade polyolefins consists in the introduction of antioxidants into the polymer chains. Antioxidants will react under UV, inducing degradation by photo-oxidation. Nevertheless the biodegradability of such systems is still controversial. We prefer to consider them as oxo-degradable polymers.

Polyolefins are resistant to hydrolysis, to oxidation and to biodegradation due to photoinitiators and stabilizers [8]. They can be made oxo-degradable by use of pro-oxidant additives. These additives are based on metal combinations, such as Mn^2+^/Mn^3+^. The polyolefin will then degrade by a free radical chain reaction. Hydroperoxides are first produced and then thermolysed or pyrolysed to give chain scission, yielding low molecular mass oxidation products with hydrophilic properties favourable to microorganisms.

### 2.2. Synthetic polymers with hydrolysable backbones

Polymers with hydrolysable backbones are susceptible to biodegradation under particular conditions. Polymers that have been developed with these properties include polyesters, polyamides, polyurethanes and polyureas, poly(amide-enamine)s, polyanhydrides [9,10].

#### 2.2.1. Aliphatic polyesters

This class is the most extensively studied class of biodegradable polymers, because of their important diversity and its synthetic versatility. A large variety of monomers can be used. Various routes leading to the development of synthetic polyesters exist and have been recently reviewed [11]. Polycondensation of difunctional monomers preferentially yields low molecular weight polymers. Ring opening polymerization is preferred when high molecular polymers are desired. Most biodegradable polyesters are prepared via ring opening polymerization of six or seven membered lactones [12].

The aliphatic polyesters are almost the only high molecular weight biodegradable compounds [9] and thus have been extensively investigated. Their hydrolysable ester bonds make them biodegradable. Aliphatic polyesters can be classified into two types according to the bonding of the constituent monomers. The first class consists of the polyhydroxyalkanoates. These are polymers synthesized from hydroxyacids, HO-R-COOH. Examples are poly(glycolic acid) or poly(lactic acid). Poly(alkene dicarboxylate)s represent the second class. They are prepared by polycondensation of diols and dicarboxylic acids. Examples are poly(butylene succinate) and poly(ethylene succinate).

*Polyglycolide (PGA):* PGA is the simplest linear aliphatic polyester. It is prepared by ring opening polymerization of a cyclic lactone, glycolide. It is highly crystalline, with a crystallinity of 45-55% and thus is not soluble in most organic solvents. It has a high melting point (220-225 °C) and a glass transition temperature of 35-40 °C [10]. PGA has excellent mechanical properties. Nevertheless its biomedical applications are limited by its low solubility and its high rate of degradation yielding acidic products. Consequently, copolymers of glycolide with caprolactone, lactide or trimethylene carbonate have been prepared for medical devices [10,13].

*Polylactide (PLA):* PLA is usually obtained from polycondensation of D- or L-lactic acid or from ring opening polymerization of lactide, a cyclic dimer of lactic acid. Two optical forms exist: D-lactide and L-lactide. The natural isomer is L-lactide and the synthetic blend is DL-lactide. Other different synthetic methods have been studied too. They have been reported in detail in [14].

PLA is a hydrophobic polymer due to the presence of –CH_3_ side groups. It is more resistant to hydrolysis than PGA because of the steric shielding effect of the methyl side groups. The typical glass transition temperature for representative commercial PLA is 63.8 °C, the elongation at break is 30.7% and the tensile strength is 32.22 MPa [15]. Regulation of the physical properties and biodegradability of PLA can be achieved by employing a hydroxy acids comonomer component or by racemization of D- and L- isomers [16]. A semi-crystalline polymer (PLLA) (crystallinity about 37%) is obtained from L-lactide whereas poly(DL-lactide) (PDLLA) is an amorphous polymer [17]. Their mechanical properties are different as are their degradation times [18]. PLLA is a hard, transparent polymer with an elongation at break of 85%-105% and a tensile strength of 45-70 MPa. It has a melting point of 170-180 °C and a glass transition temperature of 53 °C [19]. PDLLA has no melting point and a Tg around 55 °C. It shows much lower tensile strength [20]. PLA has disadvantages of brittleness and poor thermal stability.

PLA can be plasticized to improve the chain mobility and to favor its crystallization. Plasticization is realized with oligomeric acid, citrate ester or low molecular polyethylene glycol [21].

High molecular weight PLAs are obtained through ring opening polymerization. This route allows also the control of the final properties of PLA by adjusting the proportions of the two enantiomers [11]. Other routes are melt/solid state polymerization [14], solution polymerization or chain extension reaction [22]. High molecular weight PLA has better mechanical properties [23]. Different companies commercialize PLA with various ratios of D/L lactide and trade names and suppliers of different grades of PLA are listed in Table 1.

**Table 1:** Trade names and suppliers of PLA.

Trade name	Company	Country
NatureWorks^®^	Cargill Dow	USA
Galacid^®^	Galactic	Belgium
Lacea^®^	Mitsui Chem.	Japan
Lacty^®^	Shimadzu	Japan
Heplon^®^	Chronopol	USA
CPLA^®^	Dainippon Ink Chem.	Japan
Eco plastic^®^	Toyota	Japan
Treofan^®^	Treofan	Netherlands
PDLA^®^	Purac	Netherlands
Ecoloju^®^	Mitsubishi	Japan
Biomer^®^ L	Biomer	Germany

The rate of degradation of PLA depends on the degree of crystallinity. The degradation rate of PLLA is very low compared to PGA, therefore, some copolymers of lactide and glycolide have been investigated as bioresorbable implant materials [24]. The biodegradability of PLA can also be enhanced by grafting. The graft copolymerization of L-lactide onto chitosan was carried out by ring opening polymerization using a tin catalyst. The melting transition temperature and thermal stability of graft polymers increase with increasing grafting percentages. As the lactide content increases, the degradation of the graft polymer decreases [25].

*Poly(lactide-co-glycolide) (PLGA):* L-lactide and DL-lactide (L) have been used for copolymerization with glycolic acid monomers (G). Different ratios of poly(lactide-co-glycolide) have been commercially developed. Amorphous polymers are obtained for a 25L:75G monomer ratio. A copolymer with a monomer ratio of 80L:20G is semi-crystalline. When the ratio of monomer L/G increases, the degradation rate of the copolymer decreases.

*Polycaprolactone (PCL):* ε-caprolactone is a relatively cheap cyclic monomer. A semi-crystalline linear polymer is obtained from ring-opening polymerization of ε-caprolactone in presence of tin octoate catalyst [19]. PCL is soluble in a wide range of solvents. Its glass transition temperature is low, around -60 °C, and its melting point is 60 – 65 °C. PCL is a semi-rigid material at room temperature, has a modulus in the range of low-density polyethylene and high-density polyethylene, a low tensile strength of 23 MPa and a high elongation to break (more than 700%). Thanks to its low Tg, PCL is often used as a compatibilizer or as a soft block in polyurethane formulations.

Enzymes and fungi easily biodegrade PCL [9,26]. To improve the degradation rate, several copolymers with lactide or glycolide have been prepared [10]. PCL is commercially available under the trade names CAPA^®^ (from Solvay, Belgium), Tone^®^ (from Union Carbide, USA) or Celgreen^®^ (from Daicel, Japan). Possible applications in the medical field have been investigated.

*Poly(butylene succinate) (PBS) and its copolymers:* They belong to the poly(alkenedicarboxylate) family. They are obtained by polycondensation reactions of glycols, such as ethylene glycol and 1,4-butanediol, with aliphatic dicarboxylic acids, such as succinic and adipic acid [27]. They were invented in 1990 and developed by Showa High Polymer (Japan) under the trade name Bionolle^®^. EnPol^®^ is the trade name of the same class of polymers commercialized by Ire Chemical (Korea). Different poly(alkenedicarboxylate)s have been prepared: PBS, poly(ethylene succinate) (PES) and a copolymer i.e. poly(butylene succinate-co-adipate) (PBSA). PBSA is obtained by addition of adipic acid. Their molecular weights range from several tens to several hundreds of thousands. The use of a small amount of coupling agents as chain extenders allows the molecular weight to be increased [28]. Another copolymer was prepared by condensation of 1,2-ethylenediol, 1,4-butanediol with succinic and adipic acids by SK Chemicals (Korea) and commercialized under the trade name Skygreen^®^. Lunare SE^®^ trademark is another aliphatic copolyester commercialized by Nippon Shokubai (Japan). The structure of those copolymers, i.e. the nature of diacids and diols used, influences their properties [29] as well as their biodegradation rates [27].

PBS is a white crystalline thermoplastic, having a melting point of around 90 – 120 °C. Its glass transition temperature of about -45 °C to -10 °C is between the Tgs of PE and PP. The crystallization and the melting behavior of PBS have been reported in the literature [30,31]. Its mechanical properties resemble to those of polyethylene or polypropylene. Elongation at break is about 330% and tensile strength is 330 kg/cm^2^. In addition PBS has good processability, better than that of PLA and PGA [32]. Another polymer with a long chain branch has been prepared for specific applications (stretched blown bottles as well as foams) [33]. The biodegradation of the three grades are different according to the physical environment [34,35]. Because PBS suffers from insufficient biocompatibility and bioactivity for medical applications, surface modification was used to modify the PBS surface by means of plasma treatment [36].

In the case of copolyester (PBSA), polyester tensile strength decreases with the introduction of the secondary component (adipate), exhibiting a tendency similar to that of the other physical properties. PBS is the polyester with the highest tensile strength, while the copolymers PBSA (80/20) and PBSA (60/40) shows improved elongation [37].

*Poly(p-dioxanone) (PPDO):* It is a well-known aliphatic polyester having good physical properties. It is prepared by ring opening polymerisation of *p*-dioxanone. PPDO is semi-crystalline, with a low glass transition temperature in the range -10 °C to 0 °C. The properties of PPDO with different molecular weights have been investigated [38]. The increase of molecular weight can improve the thermal stability of PPDO. According to the results of rheological tests PPDO exhibits a shear-thinning behaviour. The tensile strength and modulus increase with molecular weights. PPDO has ultimate biodegradation because of the esters bonds in the polymer chains.

Many microorganisms in nature can degrade PPDO; it is then a good material for general uses. Nevertheless PPDO is more expensive than PBS. Novel biodegradable polyester was prepared by chain extension of PPDO with PBS [39]. Toluene diisocyanate was used as chain extender. Both polymers have good compatibility.

*Polycarbonate:* Poly(trimethylene carbonate) (PTMC) is obtained by ring opening polymerization of trimethylene carbonate, catalysed with diethylzinc. A high molecular weight flexible polymer was prepared, but displays poor mechanical performance [40]. Due to this property, its applications are limited and copolymers are more often used. Copolymers with glycolide and dioxanone have been prepared [10]. Poly(propylene carbonate) (PPC) is synthesized via copolymerization of propylene oxide and carbon dioxide. It has good properties such as compatibility, impact resistance. Its thermal stability and biodegradation need to be improved. A classical way is to blend it with other polymers [41]. Mitsubishi Gas Chemical Co. (Japan) commercializes a copolyester carbonate (PEC) namely poly[oligo(tetramethylene succinate)-co(tetramethylene carbonate)]. The content of carbonate inside the copolymer is changeable. The melting point of PEC is about 100-110 °C. Introducing carbonate into PTMS probably caused disorder in the crystal structure, thus lowering its melting point and increasing its susceptibility to enzymatic and microbial attacks, compared to polyolefins. The microbial degradability of PEC was confirmed to be higher than those of both of its constituents [42].

#### 2.2.2. Aromatic copolyesters

A large range of polyesters or copolyesters with aliphatic monomeric units of different sizes has been developed. Nevertheless mechanical properties of such polyesters are lower than those of non biodegradable polymers. Besides, aromatic polyesters are insensitive to hydrolytic degradation and to enzymatic or microbial attack. To improve them, aliphatic-aromatic copolyesters were made. Aliphatic-aromatic copolyesters consist in mixture of aliphatic and aromatic monomers. They are often based on terephatalic acid. Shaik [43] has presented a large range of aliphatic-aromatic copolyesters of different sizes.

The most frequently studied copolyester is poly(butylene adipate-co-terephtalate) (PBAT). Its commercial names are Ecoflex^®^, prepared by BASF (Germany), Easter Bio^®^ from Eastman Chemical (USA), Origo-Bi^®^ from Novamont (Italy). It is obtained by polycondensation between 1,4-butanediol and a mixture of adipic acid and terephtalic acid. It shows good mechanical and thermal properties at a concentration in terephtalic acid higher than 35% mol. The biodegradation rate decreases rapidly when the concentration became higher than 55% [44,45].

In 1997 Dupont (USA) launched a biodegradable copolyester resin, called Biomax^®^. It is a modified form of poly(ethylene terephtalate), with a high terephtalic acid content. It has a relatively high melting point of around 200 °C. Biodegradation of Biomax^®^ first begins with hydrolysis. Moisture fragments the polymers into small molecules which are bioassimilated and mineralized by naturally occurring microorganisms [46].

More recently, some studies have been published on various copolyesters including terephtalic acid and aliphatic acid used in biodegradable aliphatic polyesters. Lactic acid [47], glycolic acid [48] or succinic acid [49] were used to prepare novel biodegradable polymers by melt reaction. Synthesis and hydrolytic degradation of those new polymers are described.

#### 2.2.3. Polyamides and poly(ester-amide)s

Polyamides contain the same amide bound as in polypeptides. Nevertheless polyamides have a high crystallinity and strong chains interactions so that the rate of biodegradation is lower than that of polypeptides. Enzymes and microorganisms can degrade low molecular weight oligomers [9,50]. Biodegradation could be increased by the introduction of various side groups as benzyl, hydroxyl and methyl groups, through copolymerization for instance.

Copolymers with amide and ester groups are found to be readily degraded. The rate of degradation increases with increasing ester content. Aliphatic poly(ester-amide)s have been synthesized from 1.6-hexanediol, glycine and diacids with a various number of methylene groups varying from 2 to 8 [51]. All these polymers are highly crystalline.

Another series was prepared from 1,2-ethanediol, adipic acid and aminoacids, including glycine and phenylalanine [52]. In all cases, the polymers showed a high susceptibility to enzymatic degradation. The degradation rate could be controlled by modifying the phenylalanine:glycine ratio. Cameo is a poly(ester-amide) blend based on leucine or phenylalanine.

Bayer (Germany) presented in 1995 its first commercial polyester amide called Bak 1095^®^ but they stopped production in 2001. Bak 1095^®^ is based on caprolactam, butanediol and adipic acid. It has mechanical and thermal properties close to those of polyethylene [53]. High toughness and tensile strain at break are its characteristics. The crystallisation temperature of Bak 1095^®^ is 66 °C and the melting point is 125 °C. Because of its low crystallisation rate, Bak 1095^®^ is not suitable for injection moulding, so another grade, Bak 2195^®^, was launched in 1997. It was specially developed for injection moulding. Its crystallisation temperature is 130 °C and the melting point is 175 °C [46].

#### 2.2.4. Polyurethanes

Polyurethane, a unique polymeric material with a wide range of physical and chemical properties, has been extensively tailored to meet the highly diversified demands of modern technologies such as coatings, adhesives, fiber, foams, and thermoplastic elastomers [54].

Polyurethanes are prepared from three constituents: a diisocyanate, a chain extender and a polyol. They react to form a segmented polymer with alternating hard segment and soft segment. Soft segment is derived from polyols such as polyester polyols and polyether polyols. Hard segment is formed from the diisocyanate and the chain extender. The biodegradation of polyurethanes depends on the chemical nature of the segments.

The degradation can be tailored through an appropriate choice of the soft segment. Polyether-based polyurethanes are resistant to biodegradation. If the polyol is a polyester, then polyurethanes are readily biodegradable [55]. Biodegradable polyesters used are PCL, PLA and PGA [56,57]. It is assumed that the degradation rate is governed by soft segments, where esters bounds are located. The urethane bounds, which are located in hard segment, are not easily hydrolysed.

Novel biodegradable poly(ester urethane)s have thus been synthesized. The first consists of poly(L-lactic acid) and poly(butylene succinate) blocks [22]. It has been prepared via a chain extension reaction of dihydroxyl terminated PLLA and PBS prepolymers. Toluene-2,4-diisocyanate was used as chain extender. The crystallisation of the copolymer was caused by PBS segment. The extensibility of PLLA was largely improved by incorporating PBS segment.

The second is based on chitin /1,4-butane diol blends. The first step is the synthesis of a prepolymer of poly(ε-caprolactone) and 4,4-diphenylmethane diisocyanate. The prepolymer was extended with chitin and 1,4-butane diol. Different mass ratio of the two extenders has been used. When the content in chitin increased, the mechanical properties of prepolymers were improved [58,59]. Materials made of chitin have attractive advantages as the presence of chitin increases biodegradability, which offers applications in medicine.

The influence of the nature of the chain extender on biodegradability was studied only recently [60]. Introducing a chain extender with hydrolysable ester linkage allowed to the polyurethane hard segment to be degradable.

Most common isocyanates, however, are toxic, so aliphatic biocompatible diisocyanates have been used. Poly(ester urethane)s were prepared by reaction of lysine diisocyanate with polyester diols based on lactide or ε-caprolactone [61,62]. 1,4-diisocyanatobutane is another biocompatible diisocyanate.

In addition, driven by the continuous reduction in costs and the control of volatile organic compound emissions, the development of waterborne polyurethane or poly(urethane-urea) formulations has dramatically increased [63,64]. The resulting water-borne polyurethane materials present many of features related to conventional organic solvent-borne ones with the advantage of low viscosity at high molecular weight, non-toxicity, and good applicability [65]. They are more environmentally-friendly and their biodegradation is easier than that of conventional polyurethanes. Environmental protection can be better realized when the polyol is replaced with renewable sources, such as some vegetable oils, to synthesize the water-borne urethane materials. A novel waterborne polyurethane using rapeseed oil based polyol as soft segment was synthesized. The utilization of the rapeseed oil has recently become very widespread, including end products that range from margarine to a refined biodiesel fuel, and from environmentally friendly lubricants to meal for livestock. Castor oil is another vegetal oil that can also be used. Good mechanical properties of both tensile strength [9.3G (±1.5 MPa)] and elongation at break [520 (±20%)] were obtained. These waterborne polyurethanes were used to modify plasticized starch to prepare novel biodegradable materials with high performances [66,67]. PCL was also used as soft segment material to synthesize a waterborne polyurethane, which was used to plasticize starch [68].

#### 2.2.5. Polyanhydrides

An overview of polyanhydrides has been recently published [69]. Polyanhydrides are interesting biodegradable materials because they have two hydrolysable sites in the repeating unit. The degradation rate depends on the polymer backbone. Aromatic polyanhydrides will degrade slowly over a long period, while aliphatic polyanhydrides can degrade in a few days. Various routes have been investigated to their preparation: melt condensation of diacids (or diacid esters); ring opening polymerization of anhydrides; interfacial condensation; reaction of diacyl chloride with coupling agents [70].

Aliphatic homo-polyanhydrides have limited applications because of their high crystallinity and fast degradation. This is the case of poly(sebacic anhydride). The degradation rate of polyanhydride can be managed by adjusting the hydrophobic and hydrophilic components in the copolymer. Increase in the hydrophobicity of the diacid building blocks of the polymers resulted in slower degradation. Copolymers with a hydrophobic aromatic comonomer such as carboxyphenoxypropane have been extensively investigated as biomaterials [71]. Their degradation products are non-toxic and biocompatible.

As a large range of diacid monomers is available, polyanhydrides with different linkages have been developed. These include ether, ester and urethane linkages. To improve mechanical properties of polyanhydrides for specific medical applications, copolymers of anhydride with imide were also developed [72]. Their good mechanical performance has been demonstrated. Another approach is the incorporation of acrylic functional groups in the monomeric unit. This leads to photocrosslinkable polyanhydrides. The mechanical strength and degradation rate of these crosslinked polyanhydrides depend on the nature of the monomeric species.

### 2.3. Synthetic polymers with carbon backbones

Polymers with carbon backbones, such as vinyl polymers, require an oxidation process for biodegradation. Hydrolysis cannot occur.

#### 2.3.1. Vinyl polymers

Vinyl polymers are generally not susceptible to hydrolysis. An oxidation process is required for their biodegradation. Most of biodegradable vinyl polymers contain an easily oxidizable functional group and catalyst is added to promote their oxidation or photooxidation [9].

Polyvinyl alcohol is widely used because of its solubility in water. It can be easily biodegraded by microorganisms as well as enzymes [73]. It has been developed by Environmental Polymers (UK) under the trade name Depart^®^. The incorporation of photosensitive group into the polymers as ketones yields to poly(enol-ketone). They are easier hydrolysed and biodegraded than polyvinyl alcohol.

 Polyacrylates are generally resistant to biodegradation [9]. Biodegradable segments, as peptides, have been incorporated into the polymer chains yielding to biodegradable polymers. In the field of biomedical applications poly(alkylcyanoacrylate)s are extensively used. They are prepared by an anionic polymerization of alkyl cyanoacrylic monomers. A little amount of moisture is used as initiator. Those polymers are the fastest degrading polymers. Degradation time ranges from few hours to few days. It depends on the length of the alkyl side substituent. When the alkyl side group is short, very fast degradation is noticed, however degradation products are toxic. Polymers with a longer alkyl substituent are thus preferred [10].

Other biodegradable polymers have been studied especially for biomedical applications, because each of these applications requires materials with specific properties. This includes poly(ortho ester)s, poly(propylene fumarate), poly(amino acid)s, polyphosphazenes and polyphosphoesters. They are covered in another review [10].

## 3. Biodegradable Polymers Derived from Renewable Resources

Biodegradable polymers obtained from renewable resources have attracted much attention in recent years. This new interest results from global environmental respect awareness and the fossil depletion problem. Biopolymers research and development as well as their production have been the fastest for several years.

### 3.1. Natural polymers or agro-polymers

Natural polymers are formed in nature during the growth cycles of all organisms. Natural biodegradable polymers are called biopolymers. Polysaccharides, as starch and cellulose, represent the most characteristic family of these natural polymers. Other natural polymers as proteins can be used to produce biodegradable materials. These are the two main renewable sources of biopolymers. Another resource is lipids. To improve the mechanical properties of such polymers or to modify their degradation rate, natural polymers are often chemically modified.

#### 3.1.1. Proteins

Proteins are thermoplastic heteropolymers. They are constituted by both different polar and non polar α-aminoacids. Aminoacids are able to form a lot of intermolecular linkages resulting in different interactions. These offer a wide possibility of chemical functionalities and functional properties. Most of the proteins are neither soluble nor fusible, especially fibrous proteins as silk, wool and collagen [9]. So they are used in their natural form. Casting of film-forming solutions allows the preparation of films. To process protein based bioplastics, classical way is the thermoplastic processing, which consists of mixing proteins and plasticizers. Flexibility and extensibility of films are improved by the use of plasticizers [74,75]. The biodegradation of proteins is achieved by enzymes, as protease, and is an amine hydrolysis reaction. Grafting of protein is a mean to control the rate of biodegradation [9].

##### 3.1.1.1. Proteins from animal sources

Collagen is the primary protein component of animal connective tissues. Twenty two types of collagen exist. Collagen is composed of different polypeptides, which contain mostly glycine, proline, hydroxyproline and lysine. The flexibility of the collagen chain depends on the glycine content. More flexibility is obtained with an increase content of glycine [76]. Collagen is enzymatically degradable and has unique biological properties. It has been extensively investigated for biomedical applications [10].

Collagen molecules were linked onto the surface of cellulose and poly(vinyl alcohol) films via covalent bonding [77]. Activation methods using cyanogen bromide or *p*-toluenesulphonyl chloride were used. The amount of bound protein was lower for PVA than for cellulose. The cellulose film was found to become brittle and weak after activation, but the PVA films was not. Changes in degree of swelling and solubility of both activated films have been reported.

By denaturation and/or physical – chemical degradation of collagen, a high molecular weight polypeptide is produced, called gelatine [78]. Gelatine is also a protein. It consists of 19 aminoacids. It is water soluble. Gelatine has good film forming abilities. The mechanical and barrier properties of these films depend on the physical and chemical characteristics of the gelatine, especially the aminoacid composition and the molecular weight distribution. Combining gelatine with other biopolymers as soy protein, oils and fatty acids or certain polysaccharides may improve the physical properties of gelatine films [79]. Mechanical and water vapour barrier properties of gelatine films also depend on the plasticizers used [80]. Grafting was also used for the modification of gelatine [81]. Methyl methacrylate and poly(ethyl acrylate) were grafted onto gelatines by radical initiators. The composition of the graft copolymer depended on the temperature used in the process. Generally the number of branches is small whereas the molecular weight of the branches is high. It was noticed that the extent of degradation seems to decrease with increasing grafting efficiency. Proteases degrade gelatine by hydrolysing the amide function [9]. Elastin, albumine and fibrin are other proteins from animal sources. They have been investigated especially for various biomedical applications [10].

##### 3.1.1.2. Proteins from vegetal sources

Proteins derived from plants are produced at a kilo tonne per annum scale. Wheat gluten is a protein by-product of the starch fabrication. It is readily available in high quantity and at low cost. Wheat gluten contains two main groups of proteins, gliadin and glutenin. Gliadins are proteins molecules with disulphide bonds. They have low molecular weight and a low level of aminoacids with charged side groups. Glutenins are more sophisticated proteins, with a three dimensional structure. Their molecular weight is at least ten times higher than that of gliadins. Wheat gluten materials have the fastest degradation rates. Gluten is fully biodegradable and the products obtained are non-toxic.

Wheat gluten has been proven to be an excellent film forming agent. Without plasticizer, wheat gluten films are brittle [82]. The effects of water, glycerol and sorbitol on the glass transition temperature of wheat gluten were studied [83]. Compared to water, glycerol and sorbitol have higher molecular weights and lower evaporation rates, so their accessibility to various zones is limited. Thus the plasticizing effect of glycerol and sorbitol is less important than that of water. It was shown that by plasticizing gluten with glycerol, a malleable phase can be obtained [84,85]. This phase resembles a structured viscoelastic solid with pseudo-plastic behaviour. As crosslinking reactions occur at temperature higher than 60 °C, the temperature range of the use of wheat gluten is limited [82].

Soy protein: It has been used since 1959 as an ingredient in a variety of foods for its functional properties, which include emulsification and texturizing. Recently the popularity of soy protein has been increasing, mainly because of its health benefits. It has been proven that soy protein can help to prevent heart problems. According to the production method different categories of soy proteins exist: soy protein isolate, soy protein concentrate and textured soy protein. Soy protein isolate is the most refined form of soy protein and contains about 90 percent protein. Soy protein concentrate is basically soybean without the water soluble carbohydrates. It contains about 70 percent of protein. Textured soy protein, often called TSP, is made from soy protein concentrate by giving it some texture. As for soy protein concentrate, it contains about 70 percent protein [86]. Soy protein films do not have good mechanical and barrier properties as most of protein films, due to their hydrophilic nature. They are used to produce flexible and edible films.

#### 3.1.2. Polysaccharides

The principal polysaccharides used in materials applications are cellulose and starch, but other are also exploited on a lesser scale.

##### 3.1.2.1. Polysaccharides from marine sources

Chitin: It is the second most abundant natural biopolymer. It is a linear copolymer of *N*-acetyl-glucosamine and *N*-glucosamine with β-1,4 linkage. These units are randomly or block distributed throughout the biopolymer chain depending on the processing method used to obtain the biopolymer. Chitin is usually found in the shells of crabs, shrimp, crawfish and insects. It could be considered as amino cellulose. Recent advances in fermentation technology suggest that the cultivation of fungi can provide an alternative source of chitin [87]. The study reports the exploitation of both sources to produce chitin. The protein content in chitin obtained form these two methods is different. It is less than 5% for the chitin extracted from shells and reaches 10 – 15% for the chitin produced by fungi. The molecular weights for all chitin samples were in the same range. A review of chitin and chitosan has been recently published [88]. It details the distribution of chitin and chitosan in nature and the biosynthesis of chitin and chitosan by applying microorganisms. Chitinase, an enzyme, degrades chitin. As chitin has a poor solubility, it is often substituted for many applications [89].

Chitosan: Chitin is processed to chitosan by partial alkaline *N*-deacetylation. In chitosan glucosamine units are predominant. The ratio of glucosamine to acetyl glucosamine is reported as the degree of deacetylation. This degree may range from 30% to 100% depending on the preparation method and it affects the crystallinity, surface energy and degradation rate of chitosan.

Chitosan is insoluble in water and alkaline media. This is due to its rigid and compact crystalline structure and strong intra- and intermolecular hydrogen bonding. Chitosan can only soluble in few dilute acid solutions. Then chitosan is dissolved in acidic solutions before its incorporation into biodegradable films [90]. Enzymes such as chitosanase or lysozymes are known to degrade chitosan.

The applications of chitin and chitosan are limited because of their insolubility in most solvents. As chitosan has amino and hydroxyl reactive groups, chemical modifications can be proceeded. Modified chitosan have been prepared as *N*-carboxymethylchitosan or *N*-carboxyethylchitosan. They have been prepared for use in cosmetics and in wound treatment [91].

Chemical modifications of both polymers are of interest. These modifications do not change the fundamental skeleton of polymers and keep their physicochemical and biochemical properties. New properties could be introduced depending on the chemical nature of the group introduced. A lot of different derivatives have been prepared [92,93,94].

##### 3.1.2.2. Polysaccharides from vegetal sources

Starch: it is a well–known hydrocolloid biopolymer. It is a low cost polysaccharide, abundantly available and one of the cheapest biodegradable polymers. Trade names and suppliers of starch are reported in Table 2. Starch is produced by agricultural plants in the form of granules, which are hydrophilic. Starch is mainly extracted from potatoes, corn, wheat and rice. It is composed of amylose (poly-α-1,4-D-glucopyranoside), a linear and crystalline polymer and amylopectine (poly-α-1,4-D-glucopyranoside and α-1,6-D-glucopyranoside), a branched and amorphous polymer. The relative amounts and molar masses of amylose and amylopectine vary with the starch source, yielding to materials of different mechanical properties and biodegradability [95,96]. As the amylose content of starch increases, the elongation and strength increase too.

**Table 2:** Commercially available Starch and blends with polyesters.

Trade name	Company	Country
Mater-Bi^®^, Biocool^®^	Novamont	Italy
Solanyl^®^	Rodenburg Biopolymers	Netherlands
Ecofram^®^	National Starch	USA
Vegeplast^®^	Végémat	France
Biolice^®^	Limagrain	France
Biotech^®^	Biotech	Germany
Bioplast^®^	Biotec	England
Plantic^®^	Plantic Technologies	Australia

The stability of starch under stress is not high. The glucoside links start to break at 150 °C and above 250 °C the granules collapse. Retrogradation, i.e. reorganization of hydrogen bonds, is observed at low temperatures, during cooling.

In its applications starch can be either mixed, kept intact, in used in various resins as a filler or melt for blending compounds. In the former form, fillers are starch whiskers used with polymer resins. Starch nanocrystals can be obtained by partial acid hydrolysis of the amorphous regions of granules. It is then incorporated into natural polymers as PHA natural rubber or starch itself [97,98,99].

In the latter form, the molecular order inside the granules must be destroyed to improve starch processability. Granules are gelatinized in water at 130 °C. Starch is usually used as a thermoplastic. It is plasticized through destructuration in presence of specific amounts of water or plasticizers and heat and then it is extruded [100]. The most common plasticizers are polyols as glycerol [101]. When used, polyols may induce a recrystallisation reaction called retrogradation. The properties of the extruded starch depend on the water content and relative humidity [102]. Thermoplastic starch (TPS) has a high sensitivity to humidity. Thermal properties of TPS have been shown to be more influenced by the content water than the starch molecular weight [103]. TPS thus obtained is almost amorphous. A new crystalline form induced by the process can remain in the thermoplasticized product.

The plasticizer content is another important parameter. The interactions between the plasticizer and starch are weak for a plasticizer amount below 10% wt. The material is then fragile and it is difficult to work with it. When the plasticizer content becomes higher than 20% wt, flexibility and elongation properties improved [104].

Biodegradation of starch is achieved via hydrolysis at the acetal link by enzymes [9,105]. The α-1,4 link is attacked by amylases while glucosidases attack the α-1,6 link. The degradation products are not toxic.

Thermoplastic starch or plasticized starch offers an interesting alternative for synthetic polymers in specific applications. It is used for example in starch-based composites. Significant research is done in developing a new class of fully biodegradable “green” composites called biocomposites [106]. They consist in biodegradable plastics with reinforcements of biodegradable natural fibers. Starch can be used as the biodegradable polymeric compound.

However starch-based products suffer from water sensibility, brittleness and poor mechanical properties. To solve these problems various approaches are possible. They include chemical modification. Starch has two important functional groups, hydroxyl groups (-OH) and ether bonds (C-O-C). The hydroxyl group has a nucleophilic character and is susceptible to substitution reactions. To improve the mechanical properties of starch, it can be modified by acetylation [107]. Starch acetate is prepared by acetylation of starch with a mixture of pyridine and acetic acid. Casting of acetylated starch was realized from solutions of formic acid. The wet strength of the films could be maintained when the acetyl content is sufficient. The starch acetate has a high content of linear amylose and it is consequently more hydrophobic than starch. By reducing the water sensibility, mechanical properties are improved [104]. Polymers with various degrees of acetylation could be easily produced yielding to a broad range of hydrophobicities. Grafting of monomers like styrene and methylmethacrylate to the starch backbone is another strategy, but the grafted chains are not easily biodegradable [108].

Another approach to improve the mechanical properties of starch-films is blending. Starch blends with synthetic biodegradable polymers have been extensively studied [109,110]. Those systems are described in Section 4. Intensive research work has also been devoted to developing blends with nonbiodegradable polymers [8,111]. Nevertheless such systems are not considered to be biodegradable materials but may be partially biodegradable. Only the fraction of starch in the mixture which is accessible to enzymes could be degraded. Those systems are not being developed in this review.

Cellulose: it is another widely known polysaccharide produced by plants. It is a linear polymer with very long macromolecular chains of one repeating unit, cellobiose. Cellulose is crystalline, infusible and insoluble in all organic solvents [9]. Biodegradation of cellulose proceed by enzymatic oxydation, with peroxidase secreted by fungi. Cellulose can also be degraded by bacteria. As for starch degradation products are non toxic [112].

Because of its insolubility and infusibility, cellulose should be transformed to be processable. Important derivatives of cellulose are produced by reaction of one or more of the hydroxyl groups present in the repeating unit. Ethers, esters and acetals are the main derivatives. Tenite^®^ (Eastman, USA), Bioceta^®^ (Mazzucchelli, Italy), Fasal^®^ (IFA, Austria) and Natureflex^®^ (UCB, Germany) are trade names of cellulose-based polymers.

Cellulose esters are modified polysaccharides. Various degrees of substitution can be obtained. Their mechanical properties and their biodegradation decrease when the degree of substitution increases [113,114]. Cellulose acetate (CA) is one of the most important cellulose derivatives. Commercially available cellulose acetate has a degree of substitution between 1.7 and 3. Tensile strength of cellulose acetate films is comparable to polystyrene. Bioceta^®^ produced by Mazzuccheli (IT) and EnviroPlastic Z^®^ produced by Planet Polymer (US) are two of the commercially available acetate celluloses. Cellulose acetate can be obtained from agricultural by-products [115]. Agricultural products based on lignocellulose can be used for production of fuel ethanol. Lignocellulose is converted into ethanol via a four step process including enzymatic saccharification and fermentation of hemicellulosic sugars. Cellulose is then obtained as a residue. It could be used for CA preparation.

CA is currently used in fiber or film applications. CA has a high glass transition temperature, which limits its thermal processing. Most CA must be plasticized if they are used in thermoplastic applications because its decomposition temperature is lower than its melt processing temperature. [116]. An alternative way is blending CA with flexible polymers. Another method to overcome this problem is the synthesis of thermoplastic derivatives of CA by graft reaction. Different ways of graft copolymerization of cellulose diacetate onto PLA have been reported [117]. Cellulose diacetate-graft-poly(lactic acid)s were synthesized through a copolymerization of lactic acid, or through a ring-opening copolymerization of L-lactide either in dimethylsulfoxide or in bulk. All the copolymers have the same glass transition temperature of around 60 °C, close to that of PLA homopolymer. The drawability of copolymers increases a lot with increasing content of PLA. When the %wt of PLA reached 79%, the elongation at break reaches a maximum of around 2,000%.

Alginic acid or alginate: is another polysaccharide, present in brown algae. It contains carboxyl groups in each constituent residue. Alginates are extracted from the algae using a base solution. Its reaction with acid yields to alginic acid. Alginate is a non-branched, binary copolymer. It is composed of β-D-mannuronic acid monomer linked to α-L-guluronic acid monomer, through a 1,4-glycoside linkage. The ratio between the two monomers varies with the sources. Alginic acid is able to form gels in the presence of counterions, as divalent cations, such as Ca^2+^. The pH, type of counterion and the functional charge density of this polymer affect the degree of crosslinking [118]. This gelling property allows the encapsulation of various components.

Polysaccharides, such as hyaluronic acid and chondroitin sulphate, are of human origin. Their use so far has been in specific biomaterial applications [10]. They are not being described here.

### 3.2. Bacterial Polymers

They are polyesters obtained by polymerisation of monomers prepared by fermentation process (semi-synthetic polymers) or produced by a range of microorganisms, cultured under different nutrient and environmental conditions (microbial polymers) [119]. These materials are accumulated in microorganisms as storage materials.

#### 3.2.1. Semi-synthetic polymers

The fermentation of sugars produces different monomers, which are converted to polymers [120,121]. A new range of PLA was first manufactured by Cargill Dow Polymers [122]. PLA is synthesized from lactic acid produced via starch fermentation from lactic bacteria. Starch is converted into sugar which is then fermented to give lactic acid [123]. The lactic acid prepared by this biotechnological method is almost exclusively L-lactic acid [124]. PLA is completely degraded under compost conditions. PLA is not soluble in water; nevertheless microorganisms in marine environments can degrade it. PLA is a hard material. Its hardness is similar to acrylic plastic. Others characteristics have been reported in Section 2.2.1.

#### 3.2.2. Microbial polymers

Most of studies deal with poly(β-hydroxyalcanoate)s (PHA). They represent natural polyesters. Nevertheless, increasing attention is given recently to carbohydrate polymers produced by fermenting a sugar feedstock with bacteria or fungi [125,126]. They are xanthan, curdlan, pullulan and hyaluronic acid. Biosynthesis of poly(amino acid)s from microorganism was also reported [127].

##### 3.2.2.1. Microbial polyesters

In the future PHAs may be produced by plants [128] or transgenic plants [129]. A number of bacteria can accumulate PHAs as intracellular reserve materials. Some organisms accumulate PHA from 30% to 80% of their cellular dry weight, in the presence of an abundant source of carbon and under limited nitrogen [130]. The general formulae of the monomer unit is -[O-CH(R)-CH_2_-CO]-. According to the size of the alkyl substituent (R) mechanical properties of PHA differ [9,131]. Rigid brittle plastics to flexible plastics or strong tough elastomers can be obtained. PHAs are wholly biodegradable. Biodegradation occurs via linkage break by esterases of the terminal monomer from the chain ends [132].

Poly(hydroxybutyrate) (PHB): Since 1925, this polyester is produced biotechnologically and was attentively studied as biodegradable polyester [133]. The R alkyl substituent group is methyl. PHB is highly crystalline with crystallinity above 50%. Its melting temperature is 180 °C. The pure homopolymer is a brittle material. Its glass transition temperature is approximately of 55 °C. It has some mechanical properties comparable to synthetic degradable polyesters, as PLA [134]. During storage time at room temperature a secondary crystallization of the amorphous phase occurs. As a result, stress and elongation modulus increase (E = 1.7 GPa) while the polymer becomes more brittle and hard. Elongation at break is then much lower (10%) [135]. Compared to conventional plastics, it suffers from a narrow processability window [136]. PHB is susceptible to thermal degradation at temperatures in the region of the melting point [137]. To make the process easier, PHB can be plasticized, with citrate ester.

PHB is degraded by numerous microorganisms (bacteria, fungi and algae) in various environments [138]. The hydrolytic degradation yields to the formation of 3-hydroxy butyric acid, a normal constituent of blood, nevertheless with a relatively low rate.

Different monomers have been grafted onto PHB to prepare biodegradable polymers to be used for wastewater treatments. The grafted monomers were either hydrophilic as acrylic acid or sodium-p-styrene sulfonate, or hydrophobic as styrene or methyl acrylate [139]. The degree of grafting was different according to the monomers, increasing with the following order styrene, sodium-p-styrene sulfonate, methyl acrylate and acrylic acid.

Multicomponent polymeric systems containing PHB have been obtained by two ways. The first is a radical polymerization of an acrylic polymer in the presence of PHB. The second consists in melt mixing PCL with PHB. Peroxide is used in both processes to form intergrafted species responsible for compatibilization [140]. These methods have been considered as reactive blending.

It should be noted that apart from the bacterial synthetic way, other chemical ways have been developed for the production of PHB. The ring opening polymerization of β-butyrolactone yields to PHB too [141,142,143]. Different structures are obtained according to the synthesis route. An isotactic polymer with random stereosequences is obtained via bacterial process while a polymer with partially stereoregular block is obtained via chemical synthesis.

*Poly(hydroxybutyrate-co-hydroxyvalerate) (PHBV):* Among PHAs, the main biodegradable microbial polymer studied is a copolymer of hydroxybutyrate and hydroxyvalerate (HV). It was first synthesized by ICI in 1983. It can be produced by adding propionic acid to nutrient feedstock supplied to bacteria. Mixed carbon source is also used [144]. PHBV is a highly crystalline polymer with a melting point of 108 °C and glass transition temperature in the range -5 °C to 20 °C [140]. The pure copolymer is also brittle, less than PHB. Elongation at break is lower than 15% and elastic modulus is 1.2 GPa.

The melting temperature and the mechanical properties can be modified by changing the hydroxyvalerate unit content. The melting point of the copolymer, as well as the glass transition temperature and the crystallinity decrease as the hydroxyvalerate unit content increases [46,145]. The impact strength increases and the tensile strength decreases with an increase of the HV units [145,146].

The rate of degradation of PHBV is faster than that of PHB. The degradation kinetics depend on the structure (copolymer or homopolymer) and the crystallinity, and as a consequence, on the processing conditions [147].

PHB and PHBV are commercially available under different trade names: Biopol^®^ from Mosanto (USA), Nodax^®^ from Procter & Gamble (US) and Kaneka corporation (Japan), Eamat^®^ from Tianan (China) and Biomer P^®^ from Biomer (Germany).

To overcome the poor mechanical properties of PHB or PHBV and to increase the rate of degradation of those polymers, they can be mixed with other polymers or additives [148,149]. Nevertheless blends with other polymers are difficult to obtain because of chemical incompatibility. Additives can be chosen between the following list [135]: nucleating agents as saccharin, plasticizers as glycerol, triacetin or tributyrin, processing lubricants like glycerol mono(or tri)stearate. When plasticization is obtained the PHBV properties are modified [150].

PHAs are sensitive to the process conditions. A quick diminution of viscosity is obtained during the extrusion process. By increasing the shear level, the temperature and the residential time the molecular weight of PHA decreases [151].

It has also been reported that feeding the bacteria with different carbon sources led to the productions of materials with better mechanical properties [152,153]. Polyesters with longer alkyl substituent have lower degree of crystallinity, lower melting and glass transition temperatures.

Other microbial polyesters are also available. The specificity of the matrices of microorganisms is such that culture conditions, for example pH, temperature, concentration, carbon sources, and the kind of microorganisms lead to the production of various polymers [154,155]. Ninety one different hydroxyalkanoic acids have been reviewed [156]. Marine microorganisms could also be used [157]. Nevertheless only very few PHAs are available in sufficient amounts to allow their study, which limits their commercialization. PHAs are expensive when they are used alone, so they are often blended with other less expensive polymers having complementary characteristics.

## 4. Blends of Biodegradable Polymers

Mixing biopolymers or biodegradable polymers with each other can improve their intrinsic properties.

### 4.1. Starch-based blends

Starch is totally biodegradable and is an environmentally friendly material. In addition starch has a low cost. Nevertheless, since starch is highly sensible to water and has relatively poor mechanical properties compared to other petrochemical polymers, its use is limited. A solution may be to blend it with other synthetic polymers. Many biodegradable starch-based thermoplastic blends have been developed and studied extensively. A lot of research work deals with the development of blends of starch with synthetic biodegradable polymers. These blends present several advantages [15,111]. The material properties can be adjusted to the needs of the application by modifying the composition. The blending process is low cost compared to the cost of the development of new synthetic materials. These kinds of blends are intended to be more biodegradable than traditional synthetic plastics [158].

*Starch-poly(ethylene-co-vinyl alcohol) (EVOH):* Blown films were prepared from native corn starch and EVOH with different ratios. The mechanical properties depended strongly on starch and moisture content and processing. A higher extension to break and lower tensile strength and modulus were obtained when the blend was processed at a 5 °C higher temperature. Interactions between both components have been outlined thanks to differential scanning calorimetry and dynamic mechanical analysis [159].

*Starch-polyvinyl alcohol:* TPS and PVOH have excellent compatibility and their blends are of particular interest. TPS and starch can be blended at various ratios to tailor the mechanical properties of the final material. Compared to pure TPS materials, blends present improved tensile strength, elongation and processability [160,161]. Their biodegradability has been recently investigated [162]. The PVOH content has an important impact on the rate of starch degradation: increasing the amount of PVOH will decrease this rate.

*Starch-PLA:* The mechanical properties of blends of starch with PLA using conventional processes are poor due to incompatibility. An elongation increase can be achieved by using plasticizers or reacting agents during the extrusion process. Coupling agents like isocyanates have been used. The hydroxyl groups of starch could react with the isocyanate group resulting in urethane linkages and compatibilization of these systems. The effect of gelatinization of starch was also investigated [163]. It has been shown that in PLA/gelatinized starch blends, starch could be considered as a nucleating agent, resulting in an improvement of crystallinity in PLA blends and a greater superiority of mechanical properties.

Another way to improve compatibilization is to use a compatibilizer. Maleic anhydride can be used for this purpose [164]. An initiator was used to create free radicals on PLA and improved the reaction between maleic acid and PLA. The anhydride group on maleic acid could react with the hydroxyl groups present in starch. Interfacial adhesion between starch and PLA was then significantly improved. The mechanical properties obtained for PLA/starch blends compatibilized with maleic acid are higher than those obtained for virgin PLA/starch blends. A biodegradable PLA-grafted amylose copolymer has been synthesized, to be used as compatibilizer agent in starch/PLA blends [165].

*Starch – PCL:* To prepare films by using the film blowing technique, TPS was blended with PCL to adjust the rheological properties of the melt before the process [166]. Novamont (Italy) produces a class of starch blend with different synthetic components. Its trade name is Mater-Bi^®^. Four grades are available; one of them consists of PCL (Mater-Bi^®^ Z). The highest amount of starch allows the acceleration of the degradation of PCL. The behaviour of some PCL-modified starch blends has been studied [167]. The addition of modified starch leads to an increase of the Young’s modulus of PCL and a decrease in tensile strength and elongation at break values. The blend becomes less ductile [168]. Some synthetic polymers with lower biodegradabilty are used to control the rate of biodegradation according to the applications.

The modulus of blends of high-amylose corn starch (25% wt.) and PCL was 50% higher than that of PCL and the tensile strength 15% lower. The reason why good mechanical properties compared to other blends were obtained is the good dispersion of the granules in the PCL matrix. At higher starch levels a very important decrease in mechanical properties is noticed [169]. To increase the mechanical properties of PCL/starch, blends with LDPE were prepared. It has been shown that thermal properties of blends could be improved by crosslinking with organic peroxides. Bioplastics Inc. (USA) extrudes films based on such blend, which have properties similar to those of low density polyethylene. The biodegradation rate of PCL, which is very low, can be significantly increased by the presence of starch [170].

*Starch – PBS:* PBS was blended with granular corn starch [171]. By increasing the starch content it was shown that elongation at break and tensile strength decreased. The addition of starch fillers significantly improved the degradation rate.

*Starch – PHB:* Blends incorporating PHB or PHBV were previously reviewed [172]. It was shown that poly(hydroxyalkanoate)s can form miscible blends with polymers which contain an appropriate functional group i.e. capable of hydrogen bonding or donor-acceptor interactions. The effect of blending starch in PHB was also studied [150].The properties of blend films with various proportions of starch are identical. A single glass transition temperature is obtained for all the samples, which are semi-crystalline. The tensile strength was optimum for a PHB/starch ratio of 70/30 (% wt/wt) [173]. In this particular case, an advantageous cost reduction and an improvement of mechanical properties compared to pure PHB are obtained.

PHBV blends with corn starch had poor mechanical behavior because of a poor adhesion between the starch granules and the polymer matrix [174]. Biodegradation profiles of individual polymers in the blend were studied and modeled.

Wheat starch was blended with PHBV at 160 °C. The results showed that the incorporation of 50% starch led to a decrease by half in the mechanical properties of PHBV and flexibility diminished too [175]. On the contrary Young’s modulus increased by 63%. Degradation of this blend proceeded very quickly.

The studies of blends of starch with aliphatic polyesters (PCL, PBS, PHBV) showed in all cases that only a modest level of starch is possible. To improve compatibility between the starch and the aliphatic polyester, a compatibilizer was used. It contains an anhydride functional group and was incorporated onto the polyester backbone. The tensile strength obtained for such blends was close to that of synthetic polyester, only with a small amount of compatibilizer [176].

### 4.2. Others blends

PHB or PHBV are brittle polymers. To improve their mechanical properties they are mixed with other biodegradable materials. When nucleating agents are added, smaller spherulites are formed, thus the mechanical properties are improved. In addition these properties depend on the processing conditions, morphology, crystallinity and glass temperature transition [135].

Another class of biodegradable PHB can be prepared by blending a basic PHB with cellulose esters [177]. Blends of PHBV and cellulose acetate butyrate were prepared by thermal compounding. The thermal process did not induce transesterification, nor molecular weight changes. The structure and mechanical properties depended on the PHBV content. When the PHBV content is lower than 50%, blends are amorphous, while with a higher content they become semi-crystalline. At this high content PHBV is partially miscible with cellulose acetate butyrate. These authors also studied blends of cellulose acetate propionate with poly(tetramethylene glutarate) [116]. A range of 50 to 90% wt of cellulose acetate propionate was investigated. The same process was used. The blends are amorphous.

Blends of PHBV and PPC were also studied. The crystallinity and morphology of those blends have been reported [178]. A transesterification reaction between PHBV and PPC has been outlined. The melting temperature of the blend composed of 70%wt PPC was 4 °C lower than that of pure PHBV, and the crystallization temperature decreased by about 8 °C. The effect of polyvinyl acetate (PVAc) used as compatibilizer on the thermal behavior and the mechanical properties of those blends was reported [179]. The melting point and the crystallization temperature of PHBV in blends decrease with the increase content of PVAc. Morphology analysis shows that the addition of the compatibilizer can decrease the size of dispersed phase. Young’s modulus, elongation at break and tensile strength increase in the presence of PVAc. The degradation of those blends in soil suspension was recently investigated [41]. Enzymes will preferentially degrade PHBV whereas PPC is degraded by hydrolysis.

Studies concerning blends containing PHA have been summarized in a review [172]. PHAs can form miscible blends with other polymers containing appropriate functional groups. The miscibility is obtained through hydrogen bonding or donor-acceptor interactions.

Many studies have been reported on PLA blends with various polymers. In most of systems, PLA and other polymers are immiscible. It is essential to compatibilize such blends to have good properties. Blends of PLA with PCL were prepared by melt blending and using *in situ* reactions. In this case an ester exchange reaction by alcoholysis was described [180]. In EVOH/PLA blends, the hydroxyl groups of EVOH could react with the carboxyl group of PLA through an esterification reaction in the presence of catalyst [181]. Reactive blending of PLA with ethylene copolymer gave an important improvement of mechanical properties of PLA. This was attributed to an interfacial reaction between the components [182].

Poly(aspartic acid-co-lactide) (PAL) was used to blend various polymers such as PLLA, PBS and PCL [183] in order to increase their biodegradability i.e. their degradation rate. In the case of PLLA, the mechanical properties of such blends were similar to that of non-blended PLLA but the hydrolysis rate of the PLLA was effectively enhanced. In the case of PCL melt blended with PAL, a sufficient percentage of poly(aspartic acid-co-lactide) to have an increase in degradation rate is 20% [184]. PAL was shown to improve the thermal stability of PLA in PAL/PLA blend films.

PCL blends with chitin were prepared as biodegradable composites by melt blending [185]. Increasing the amount of chitin has no effect on the melting or crystallization temperature. This was attributed to a non miscible blend. Another blending route is solvent casting [186]. The degree of crystallinity of PCL decreases upon blending with chitin. Same results are obtained with PCL/chitosan blends. These blends are expected to have good mechanical properties.

## 5. Applications

Biodegradable polymers can be processed by most conventional plastics processing techniques, with some adjustments of processing conditions and modifications of machinery. Film extrusion, injection moulding, blow moulding, thermoforming are some of the processing techniques used. The three main sectors where biodegradable polymers have been introduced include medicine, packaging and agriculture. Biodegradable polymers applications include not only pharmacological devices, as matrices for enzyme immobilization and controlled-release devices [187] but also therapeutic devices, as temporary prostheses, porous structure for tissue engineering.

As biopolymers have a low solubility in water and a very important water uptake, they could be used as absorbent materials in horticulture, healthcare and agricultural applications [188]. Packaging waste has caused increasing environmental concerns. The development of biodegradable packaging materials has received increasing attention [189]. Table 3 regroups some materials and their use.

### 5.1. Medicine and pharmacy

Biodegradable polymers used as biomaterials have been recently reviewed [7,10,190]. To be used as biomaterials, biodegradable polymers should have three important properties: biocompatibility, bioabsorbility and mechanical resistance. The use of enzymatically degradable natural polymers, as proteins or polysaccharides, in biomedical applications began thousand of years ago whereas the application of synthetic biodegradable polymers dates back some fifty years.

Current applications of biodegradable polymers include surgical implants in vascular or orthopaedic surgery and plain membranes. Biodegradable polyesters are widely employed as porous structure in tissue engineering because they typically have good strength and an adjustable degradation speed [191,192]. In these papers, the polymers are described in terms of their chemical composition, breakdown products and mechanism of breakdown, mechanical properties, and clinical limitations.

**Table 3:** Some applications of biodegradable polymers.

Product	Society	Composition	Applications
Mater-Bi^®^	Novamont (Italy)	Starch and polyester	Collection bags for green waste, agricultural films, disposable items.
Polynat^®^	Roverc’h (France)	Rye flower (80%)	Disposable items, flower containers
Ecofoam^®^	American Excelsior Company (USA)	Starch	Wrapping plastics
Biopol^®^	Goodfellow (Great Britain)	PHB/PHV	Razors, bottles
Eco-pla^®^	Cargill Dow (USA)	PLA	Sanitary products, sport clothes, conditioning and packaging
Bio-D^®^	Cirad (France)	Proteins extracted from cotton seed	Agricultural films
Ecoflex^®^	BASF (Germany)	Co-polyester	Agricultural films
Eastar Bio^®^	Eastman (Great Britain)	Co-polyester	Agricultural films
BAK 1095^®^	Bayer (Germany)	Polyester amide	Disposable items, flower containers

Biodegradable polymers are also used as implantable matrices for the controlled release of drugs inside the body or as absorbable sutures. Some commercial biodegradable medical products and their applications have been listed previously [13].

#### 5.1.1. Natural or bacterial polymers

Medical applications of biopolymers have been reported in a previous paper [193]. Proteins are the major components of many tissues and thus they have been extensively used as biomaterials for sutures, haemostatic agents, scaffolds for tissue engineering and drug delivery systems. Gelatin was used for coatings and microencapsulating various drugs for biomedical applications [194]. It has also been employed for preparing biodegradable hydrogels [195].

Chitin and its derivatives have been used as drug carriers and anti-cholesterolemic agents, blood anticoagulants, anti-tumor products and immunoadjuvants [10,196]. More recently some studies have shown the anti-oxidative and radical scavenging activities of chitosans. As a matter of fact, a primary actor in various degenerative diseases as well as in the normal process of aging is oxidative stress, induced by oxygen radicals [80,197,198]. Chitin, collagen and poly-L-leucine have been used to prepare skin substitutes or wound dressing [199]. Alginate gels have been extensively used in controlled release drug delivery systems [118]. Herbicides, microorganisms and cells have been encapsulated by alginates.

PHB and PHBV are soluble in a wide range of solvents and can be process in various shapes. Nevertheless, as PHB is brittle, its application in biomaterials is limited. As PHBV is less brittle it is potentially usable. In addition PHBV has the unique property of being piezoelectric. It is used in applications where electrical simulation is applied [10]. PHB has the advantageous property of being degraded in D-3-hydroxybutyrate, a natural constituent of human blood. As a consequence, PHB is suitable for biomedical applications. It is used in drug carriers and tissue engineering scaffolds [154,200,201].

#### 5.1.2. Synthetic polymers

Synthetic polymers are widely used in biomedical implants and devices because they can be fabricated into various shapes. In this area interest in biodegradable polymers has increased. PGA and PLA can be considered as the first biodegradable polymers used in biomedical applications. Due to their good mechanical properties, PGA and PLLA have been used as bone internal fixation devices. They also have excellent fiber forming properties and thus PGA was used to prepare absorbable sutures and PLLA to replace ligament and non-degradable fibers. Non woven PGA fabrics have been investigated as scaffolding matrices for tissue regeneration [10]. As PDLLA has lower mechanical properties and faster degradation rate than PLLA, it is often used in drug delivery systems and scaffolding matrices for tissue engineering. PLGA has shown to have a good cell adhesion and could be used for tissue engineering applications [202]. PLGA is used as polymeric shell in nanoparticules used as drug delivery systems.

Other polyesters like PBS, PPDO, PCL and their copolymers are also utilized as biomedical materials [13]. PCL is used as a matrix in controlled release systems for drugs, especially those with longer working lifetimes [203]. PCL has a good biocompatibility and is used as scaffolds for tissue engineering. PBS is a promising substance for bone and cartilage repair [36]. Its processability is better than that of PGA or PLA. It has higher mechanical properties than PE or PP. Its insufficient biocompatibility could be enhanced by plasma treatment. PPDO was used to prepare the first monofilament sutures. They have a lower risk of infection when used and are thus more interesting than multifilaments. PPDO was also used as fixation screws for bones [204].

Polyurethanes and poly(ether urethane)s have good biocompatibility and mechanical properties and have thus been used as medical implants. Polyanhydrides have been investigated in controlled release devices for drugs treating eyes disorder. They have been used as chemotherapeutic agents, local anesthetics, anticoagulants, neuro-active drugs and anticancer agents [72].

### 5.2. Packaging

In everyday life, packaging is another important area where biodegradable polymers are used. In order to reduce the volume of waste, biodegradable polymers are often used. Besides their biodegradability, biopolymers have other characteristics as air permeability, low temperature sealability and so on [189]. Biodegradable polymers used in packaging require different physical characteristics, depending on the product to be packaged and the store conditions.

Due to its availability and its low price compared to other biodegradable polyesters, PLA is used for lawn waste bags. In addition, PLA has a medium permeability level to water vapor and oxygen. It is thus developed in packaging applications such as cups, bottles, films [18,205]. PCL finds applications in environment e.g. soft compostable packaging.

Several polysaccharide-based biopolymers such as starch, pullulan and chitosan, have been investigated as packaging films. Starch films have low permeability and are thus attractive materials for food packaging. When composed of proteins and polysaccharides, the films have good mechanical and optical properties, but they are very sensitive to moisture. They also have poor water barrier properties. When composed of lipids, films are more resistant to moisture. The problem is their opacity. Moreover they are sensitive to oxidation. The current trend in food packaging is thus the use mixtures of different biopolymers [206]. Chitosan was used in paper-based packaging as a coating, to produce an oil barrier packaging [207]. Results showed that chitosan coatings can be used as fat barriers, but the treatment cost was relatively high compared to the fluorinated coatings usually used. Chitosan based films have proven to be effective in food preservation and can be potentially used as antimicrobial packaging [208].

PHB has been used in small disposable products and in packing materials [209]. Bucci [210] investigated the use of PHB in food packaging, comparing it to PP. The deformation value of PHB was about 50% lower than that of PP. PHB is more rigid and less flexible than PP. The performances of PHB tend to be lower than those of PP under normal freezing conditions. Nevertheless at higher temperatures PHB performed better than PP.

In 2002, DuPont and EarthShell commercialized food packaging and containers composed of starch and Biomax^®^. APACK^®^ is a thermoformed packaging based on starch, from Switzerland.

### 5.3. Agriculture

For this application, the most important property of biodegradable polymers is in fact their biodegradability [211]. Starch-based polymers are the most used biopolymers in this area. They meet the biodegradability criteria and have a sufficient life time to act.

Plastic films were first introduced for greenhouse coverings fumigation and mulching in the 1930s. Young plants are susceptible to frost and must be covered. The main actions of biodegradable cover films are to conserve the moisture, to increase soil temperature and to reduce weeds in order to improve the rate of growth in plants. At the end of the season, the film can be left into the soil, where it is biodegraded [212]. Another application bases on the production of bands of sowing. It is bands which contain seeds regularly distributed as well as nutriments. In the field of geotextiles, we can mention the use of textiles based on biopolymers for filtration and drainage and the use of the geogrilles [213].

Biodegradable polymers can be used for the controlled release of agricultural chemicals. The active agent can either be dissolved, dispersed or encapsulated by the polymer matrix or coating, or is a part of the macromolecular backbone or pendent side chain. The agricultural chemicals concerned are pesticides and nutrients, fertilizer, pheromones to repel insects. The natural polymers used in controlled release systems are typically starch, cellulose, chitin, aliginic acid and lignin [9].

In horticulture threads, clips, staples, bags of fertilizer, envelopes of ensilage and trays with seeds are applications mentioned for biopolymers. Containers such as biodegradable plant pots and disposable composting containers and bags are other agricultural applications. The pots are seeded directly in the soil, and break down as the plant begins to grow.

In marine agriculture, biopolymers are used to make ropes and fishing nets. They are also used as support for the marine cultures [214].

In mulching and low-tunnel cultivation, to enhance sustainability and environmentally friendly agricultural activities, a promising alternative is the use of biodegradable materials. Agricultural films placed in the soil are susceptible to ageing and degradation during their useful lifetime, so they need to have some specific properties [15].

When starch is placed in contact with soil microorganisms, it degrades into non toxic products. This is the reason why starch films are used as agricultural mulch films. Mater-Bi based biodegradable films were developed and tested [148]. Water and high temperatures do not affect the mechanical behavior of the biodegradable films. Negative effects on the elongation at break were obtained with a high dose of UV radiation.

### 5.4. Others fields

Biopolymers are also used in shape specific applications such as in the automotive, electronics or construction sectors.

*Automotive:* The automotive sector aims to prepare lighter cars by use of bioplastics and biocomposites. Natural fibers can replace glass fibers as reinforcement materials in plastic car parts [215]. We await the development of the bio-composite materials. For example the PLA is mixed with fibers of kenaf for replace the panels of car doors and dashboards (Toyota Internet site). Starch-based polymers are used as additive in the manufacturing of tires. It reduces the resistance to the movement and the consumption of fuel and in fine greenhouse gas emissions (Novamont Internet site).

*Electronics:* PLA and kenaf are used as composite in electronics applications. Compact disks based on PLA are also launched on the market by the Pioneer and Sanyo groups. Fujitsu Company has launched a computer case made of PLA [216].

*Construction:* PLA fiber is used for the padding and the paving stones of carpet. Its inflammability, lower than that of the synthetic fibers, offers more security. Its antibacterial and antifungal properties avoid allergy problems . The fiber is also resistant to UV radiation.

*Sports and leisure:* Some fishing hooks and biodegradable golf tees (Vegeplast, France) are based on starch. PLA fiber is used for sports clothes. It combines the comfort of the natural fibers and the resistance of synthetic fibers.

*Biotechnological applications:* Chitin acts as an absorbent for heavy and radioactive metals, useful in wastewater treatment.

*Applications with short-term life character and disposability:* Aliphatic polyesters like PLA, PBS, PCL and their copolymers are used as biodegradable plastics for disposable consumer products, like disposable food service items (disposable cutlery and plates, for example). Other products are diapers, cotton stalk and sanitary products.

*Unusual applications:* There are a lot of other applications which do not fit into any of the previous categories. Thus combs, pens (Begreen^®^ from Pilot Pen or Green Pen^®^ from Yokozuna), and mouse pads made of biodegradable polymers have also been invented, mostly for use as marketing tools. Biodegradable polymers can be used to modify food textures. Due to its non toxicity, alginate has been used as a food additive and a thickener in salad dressings and ice creams. Chitin and chitosan are used as food and feed additives [217]. PLA (semi-synthetic polymers) is used for compostable food.

## 6. Conclusions

Biodegradable polymers have received much more attention in the last decades due their potential applications in the fields related to environmental protection and the maintenance of physical health. At present only few groups of the mentioned biopolymers are of market importance. The main reason is their price level, which is not yet competitive. The future of each biopolymer is dependent not only on its competitiveness but also on the society ability to pay for it. The future outlook for development in the field of biopolymers materials is promising.

To improve the properties of biodegradable polymers, a lot of methods have been developed, such as random and block copolymerization or grafting. These methods improve both the biodegradation rate and the mechanical properties of the final products. Physical blending is another route to prepare biodegradable materials with different morphologies and physical characteristics.

To provide added value to biodegradable polymers, some advanced technologies have been applied. They include active packaging technology and natural fiber reinforcements. Recently different studies have been reported concerning the use of nanoclay with biodegradable polymers, especially with starch and aliphatic polyesters. Nano-biocomposites or bio-nanocomposites are under investigation.
